# Caricaturing faces to improve identity recognition in low vision simulations: How effective is current-generation automatic assignment of landmark points?

**DOI:** 10.1371/journal.pone.0204361

**Published:** 2018-10-04

**Authors:** Elinor McKone, Rachel A. Robbins, Xuming He, Nick Barnes

**Affiliations:** 1 Research School of Psychology, and ARC Centre of Excellence in Cognition and its Disorders, The Australian National University, Canberra, Australian Capital Territory, Australia; 2 Research School of Psychology, The Australian National University, Canberra, Australian Capital Territory, Australia; 3 School of Information Science and Technology, ShanghaiTech University, Shanghai, China; 4 Research School of Engineering, Australian National University, Canberra, Australian Capital Territory, Australia; 5 Data61, Commonwealth Scientific and Industrial Research Organisation (CSIRO), Canberra, Australian Capital Territory, Australia; 6 Bionic Vision Australia, Carlton, Victoria, Australia; University of Nevada Reno, UNITED STATES

## Abstract

**Purpose:**

Previous behavioural studies demonstrate that face caricaturing can provide an effective image enhancement method for improving poor face identity perception in low vision simulations (e.g., age-related macular degeneration, bionic eye). To translate caricaturing usefully to patients, assignment of the multiple face landmark points needed to produce the caricatures needs to be fully automatised. Recent development in computer science allows automatic face landmark detection of 68 points in real time and in multiple viewpoints. However, previous demonstrations of the behavioural effectiveness of caricaturing have used higher-precision caricatures with 147 landmark points per face, assigned by hand. Here, we test the effectiveness of the auto-assigned 68-point caricatures. We also compare this to the hand-assigned 147-point caricatures.

**Method:**

We assessed human perception of how different in identity pairs of faces appear, when veridical (uncaricatured), caricatured with 68-points, and caricatured with 147-points. Across two experiments, we tested two types of low-vision images: a simulation of blur, as experienced in macular degeneration (testing two blur levels); and a simulation of the phosphenised images seen in prosthetic vision (at three resolutions).

**Results:**

The 68-point caricatures produced significant improvements in identity discrimination relative to veridical. They were approximately 50% as effective as the 147-point caricatures.

**Conclusion:**

Realistic translation to patients (e.g., via real time caricaturing with the enhanced signal sent to smart glasses or visual prosthetic) is approaching feasibility. For maximum effectiveness software needs to be able to assign landmark points tracing out all details of feature and face shape, to produce high-precision caricatures.

## Introduction

**Fig 1:**
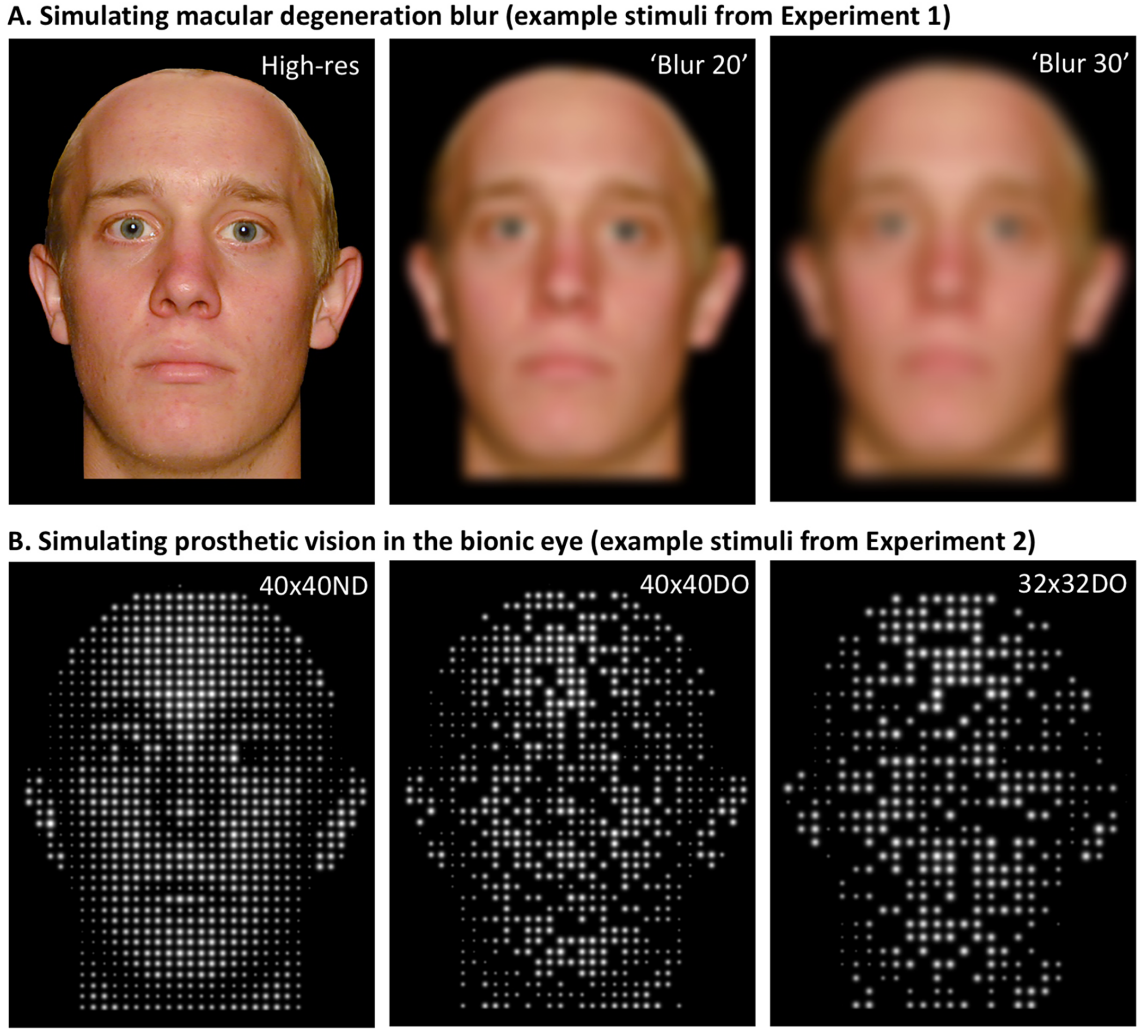
Simulations of facial appearance in low vision. **A**. High resolution face and then the same face blurred to simulate peripheral-vision blur, as relevant to blur experienced in macular degeneration. The three resolutions illustrated are those tested in Experiment 1. **B**. The same face phosphenized to simulate appearance in prosthetic vision (bionic eye). The three resolutions illustrated are those tested in Experiment 2 (ND = no electrode dropout; DO = random 30% electrode dropout).

Low-resolution vision occurs in many eye conditions, including age-related macular degeneration [1–3] and prosthetic vision (the ‘bionic eye ’ [4–7]) (Fig 1). In low vision, the ability to recognise individual faces is poor (e.g., poor ability to tell apart a set of young adult Caucasian males with short hair) [1–3,8,9]. Poor identity recognition is associated with significant difficulties in real-world social interaction [10,11], and thus it is important to develop techniques that can improve face identity perception. Image enhancement offers potential for this purpose [8,12–16].

**Fig 2:**
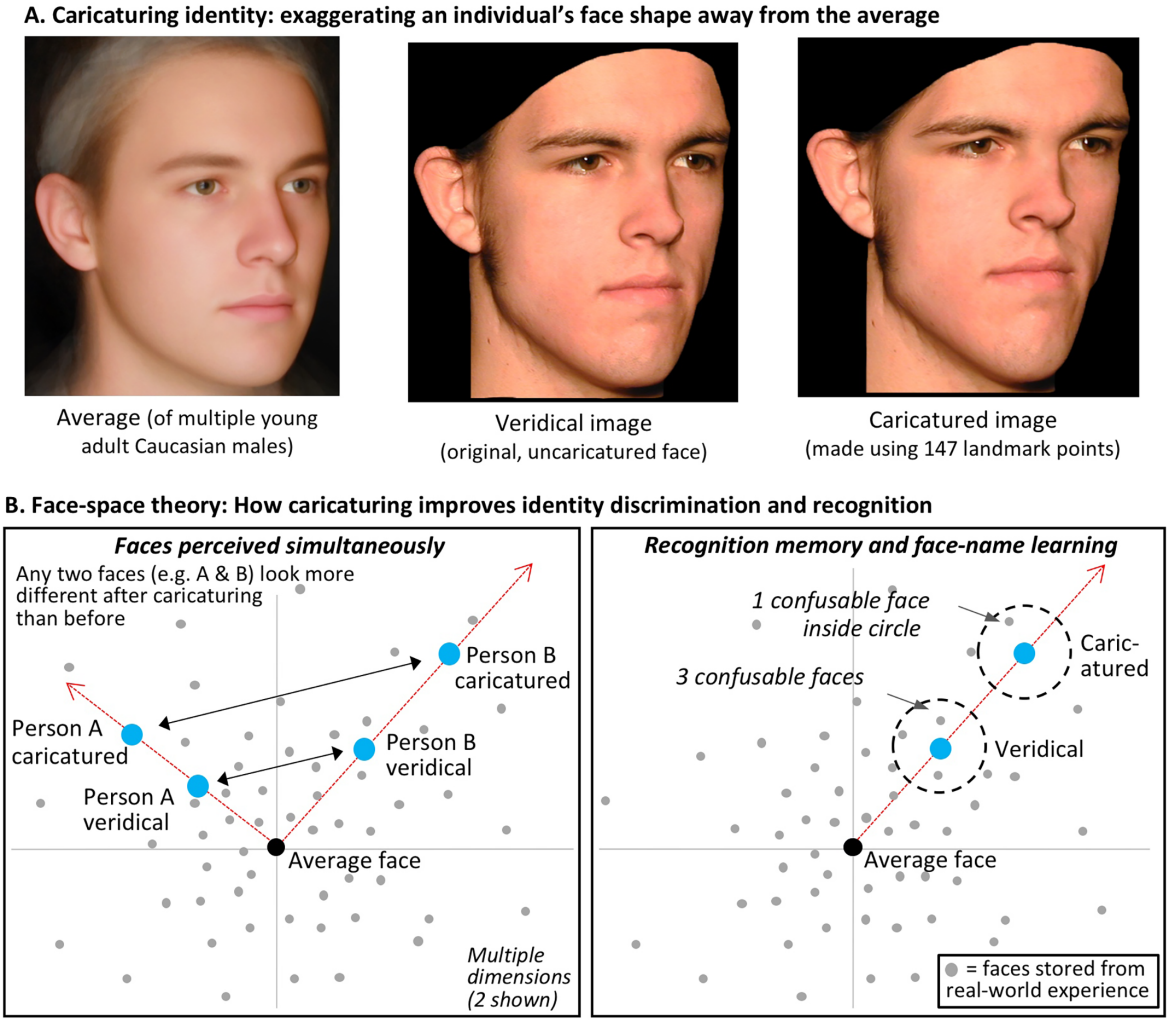
Caricaturing and face-space theory. ** A**. Caricaturing illustrated using one of our face stimuli. The shape information in the veridical (original) face is physically altered to exaggerate it away from the average face (matched to the veridical on sex, race, age group and viewpoint, to ensure caricaturing of only identity information). Note how caricaturing makes the man’s longer-than-average chin become even longer, the smaller-than-average eyebrow-to-eye distance become even smaller, the slightly turned up nose become more so, and so on. **B**. Standard explanation (for review see [29]) of caricaturing benefits in terms of a multidimensional mental face-space. The dimensions coded on the axes remain unknown (but might represent, for example, attributes such as lip thickness or width of the face).

One method of image enhancement shown to be effective is *caricaturing* of face identity. Caricaturing exaggerates the distinctive ways in which an individual person’s face shape differs from the average face, such that, for example, a naturally long nose becomes even longer, or naturally close together eyes become even closer together (see Fig 2A). It is well established that caricaturing can improve identity processing for high-resolution faces, in a wide range of tasks assessing both perception and memory [8, 17–23]. Theoretically, the standard explanation of caricature improvements is in terms of the human perceiver’s mental ‘face-space’ (Fig 2B [17,24]). Face-space has several key properties, established by multiple methods (e.g., distinctiveness effects on face memory [25,26], multidimensional scaling of pairwise similarity ratings [17,27], and Dan-antiDan face identity adaptation aftereffects [28]; for recent review see [29]). These key properties include: the centre of the space is the average face derived from the "diet" of faces to which the observer has been exposed in everyday life; the space is multidimensional, with the exact information coded on the axes still unknown but covering all important ways in which individual faces differ from each other; typical faces lie near the centre while distinctive faces lie further out; and the density of exemplars in face space decreases as one moves away from the centre [29].

Caricaturing is then explained as shifting the face along a vector away from the average face at the centre (Fig 2B). Staying on this vector retains perceived identity, making a person look like a "more distinctive version of themselves". It also improves both face discrimination and face memory. Face-discrimination is improved (Fig 2B) because caricaturing shifts any face further away from all other faces, meaning that any two (or more) people seen together will look more different from each other when caricatured than when veridical (i.e., uncaricatured). Face memory is also improved (Fig 2B) because caricaturing moves the face into a region of lower exemplar density, meaning that there are fewer nearby neighbours with which the target can be confused.

Our own lab’s research has focussed on exploring the potential for caricaturing to benefit low-resolution face recognition. We have established significant caricature improvements across multiple settings. To date, these include: (a) blurred faces [8,23], noting blur is a key property of the low-resolution vision experienced in macular degeneration [30]; (b) simulations of prosthetic vision (both with and without allowing scanning across the image) [9], (c) a wide range of resolutions (i.e., multiple blur levels or bionic eye resolutions) [8,9], (d) in young-adult and elderly observers [23], (e) for faces of one’s own race and of other, less familiar, races [23], and (f) in multiple tasks of everyday relevance, including dissimilarity ratings in simultaneous perception (as relevant to telling apart two people seen at once), old-new recognition memory (knowing whether someone has been seen before or not), and face-name learning [8,9,23]. This broad generalisability of caricature benefits derives theoretically from an origin within face-space coding in mid- and/or high-level cortical vision (e.g., V4 and/or areas in inferotemporal cortex) [9], rather than in early visual processing which would be sensitive to the specific details of the type of low vision.

Given these laboratory demonstrations of the potential wide applicability of caricaturing, our long-term aim is to translat this to technology that can be used by patients in everyday life. The goal is to eventually be able to caricature all faces a patient sees (after first isolating the face from the natural complex background [31]), with the caricatured versions fed to the patient via smart glasses (e.g., in macular degeneration) or their prosthetic implant (in patients fitted with bionic eyes).

**Fig 3:**
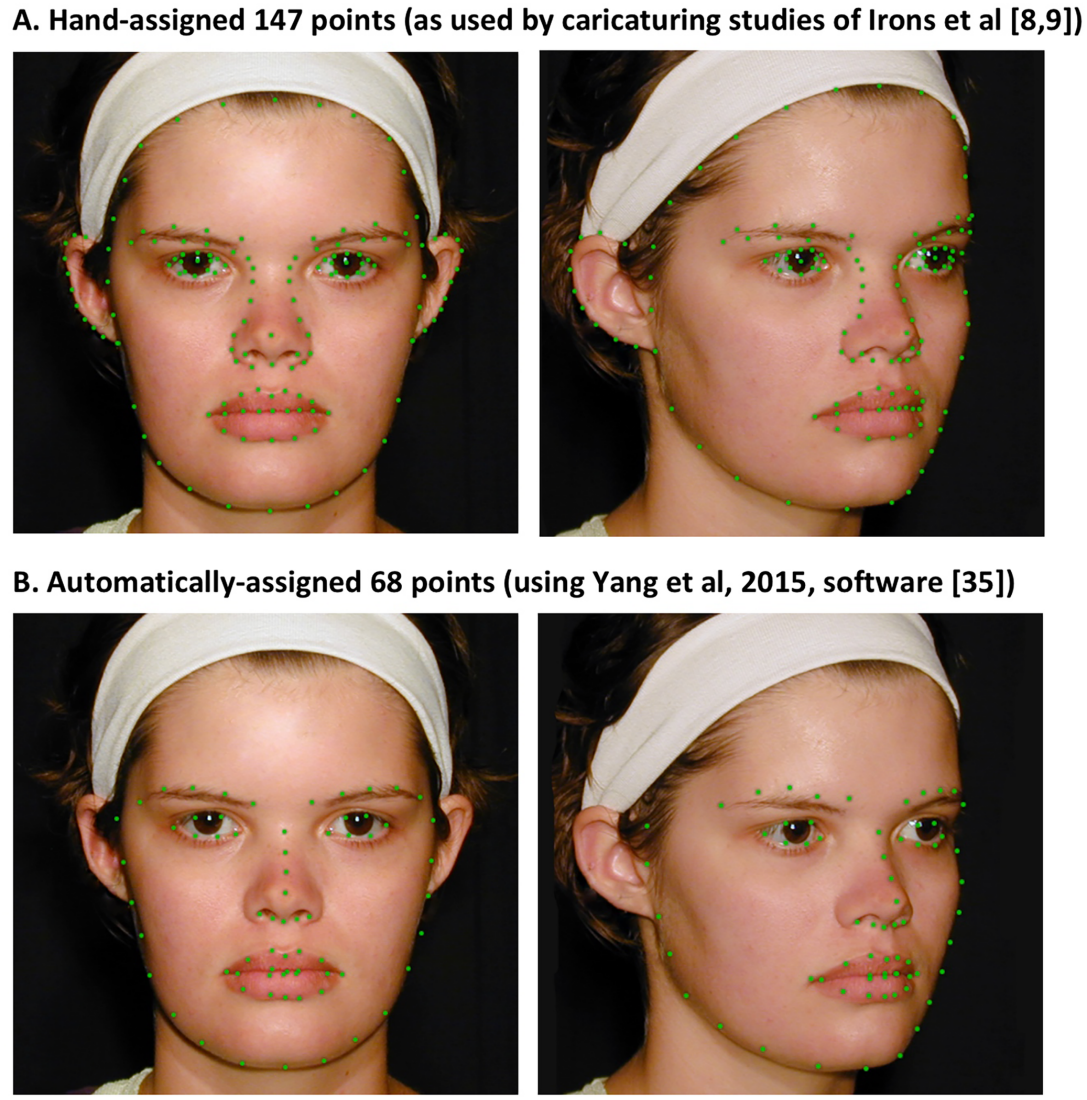
Landmark point assignment. The location of landmark points (green dots) for: **A**. 147 points per face assigned by hand as used in previous studies demonstrating caricature benefits for low-vision simulations; **B**. 68 points per face assigned automatically by the software of Yang et al (2015) [35].

For this to work, the process needs to be fully automated. While the morphing of the face images that comprises the actual caricaturing stage has been automated for decades [18], producing an accurate caricature also requires, as a first step, the assignment of multiple landmark points to the face (Fig 3). Our previous demonstrations of the effectiveness of caricaturing [8,9,23] have all used hand-assignment of landmark points (as is usual in behavioural work on caricaturing [17,18,32]). Specifically, we hand-assigned 147 points to each face, with the number and locations selected to be sufficient to trace out the detailed shape of every facial feature and also including all of the external outline of the face (Fig 3A). This level of detail produces highly precise caricatures. These caricatures resulted in a caricature benefit large enough to make the method of practical value (e.g., 6–14% improvement in recognition accuracy when task difficulty is set such that performance is half-way between chance and perfect [8,9,23]). The downside, however, is that hand-assignment is far too slow for practical applicability to patients: creating the set of 26 caricatured faces used in our previous articles took approximately 3 months of work full-time for one person.

Clearly, development and validation of automatic-assignment of landmark points is needed. Facial landmark detection has been an active research topic over past decades in computer vision [33,34]. Most existing approaches rely on machine learning techniques to train a landmark detector based on image features learned from a large human-annotated face database. Recently, however, Yang et al [35] developed a facial landmark estimation method that operates without human input, and can be applied not only to single images but also video streams in near real time. It determines if and where there is a face in the image, assigns landmark points to the face image at a speed of roughly 15 frames per second on a 3.3GHz CPU machine. It can also deal successfully with moderate viewpoint changes (up to 30° rotation away from front-view), and with partial occlusion of the face (e.g., by glasses, beards, or hands moving in front of the face). These attributes place the above method as a serious contender for offering a genuinely useful landmark assignment procedure to support caricaturing for patients in the real world.

Currently, however, the Yang et al [35] software assigns only 68 landmark points to each face, in specified locations illustrated in Fig 3B. Compared to our hand assignment [8] it has lower precision: for example, it traces out the eyes and mouth slightly less exactly, and does not cover some aspects of the face at all, notably forehead shape, ears, width of the eyebrows, or width of the middle section of the nose. (Similarly, even the best commercial software, using machine learning, does not cover the full faces due to regions not having been annotated in the available training databases; e.g., Face++, as described in their Application Programming Interface).

Here, we test whether this reduced number of landmark points is still sufficient for caricaturing to improve human perception of facial identity, and also how effective it is compared to using our larger number of hand-assigned points. Currently, little is known about the exactness of coding in humans’ perceptual face-space. If the coding is extremely precise, we might find that the 68-point caricatures would produce no benefit; theoretically, this would be because these caricatures would not shift the veridical face precisely enough on a vector away from the centre of the space to ensure it is shifted away from all other possible individuals. At the other extreme, if face-space coding is sufficiently fuzzy (i.e., has large tolerance in the coding) or uses only limited face-shape information that all happens to be captured in the Yang et al [35] landmark locations (e.g., face-space codes the location of the centre of the nose but not the width at its middle), then we might find that 68-point caricatures are as effective at improving identity discrimination as the 147-point caricatures.

We tested normal-vision observers shown low-resolution images. Experiment 1 used blurred images (Fig 1A), simulating the feature of visual appearance most commonly reported by patients with macular degeneration. Experiment 2 used phosphenised images (Fig 1B) to simulate a bionic eye, noting that implanted patients report seeing separated fuzzy balls of light (i.e., ‘phosphenes’ [36]). Each experiment tested three caricature conditions—uncaricatured (Veridical faces, *V*), caricatured based on 68 automatically-assigned landmark points (at 60% caricature strength, *C-68p*), and caricatured based on our previous 147 hand-assigned landmark points (also at 60% caricature strength, *C-147p*)—crossed with three resolution conditions (Fig 1).

**Fig 4:**
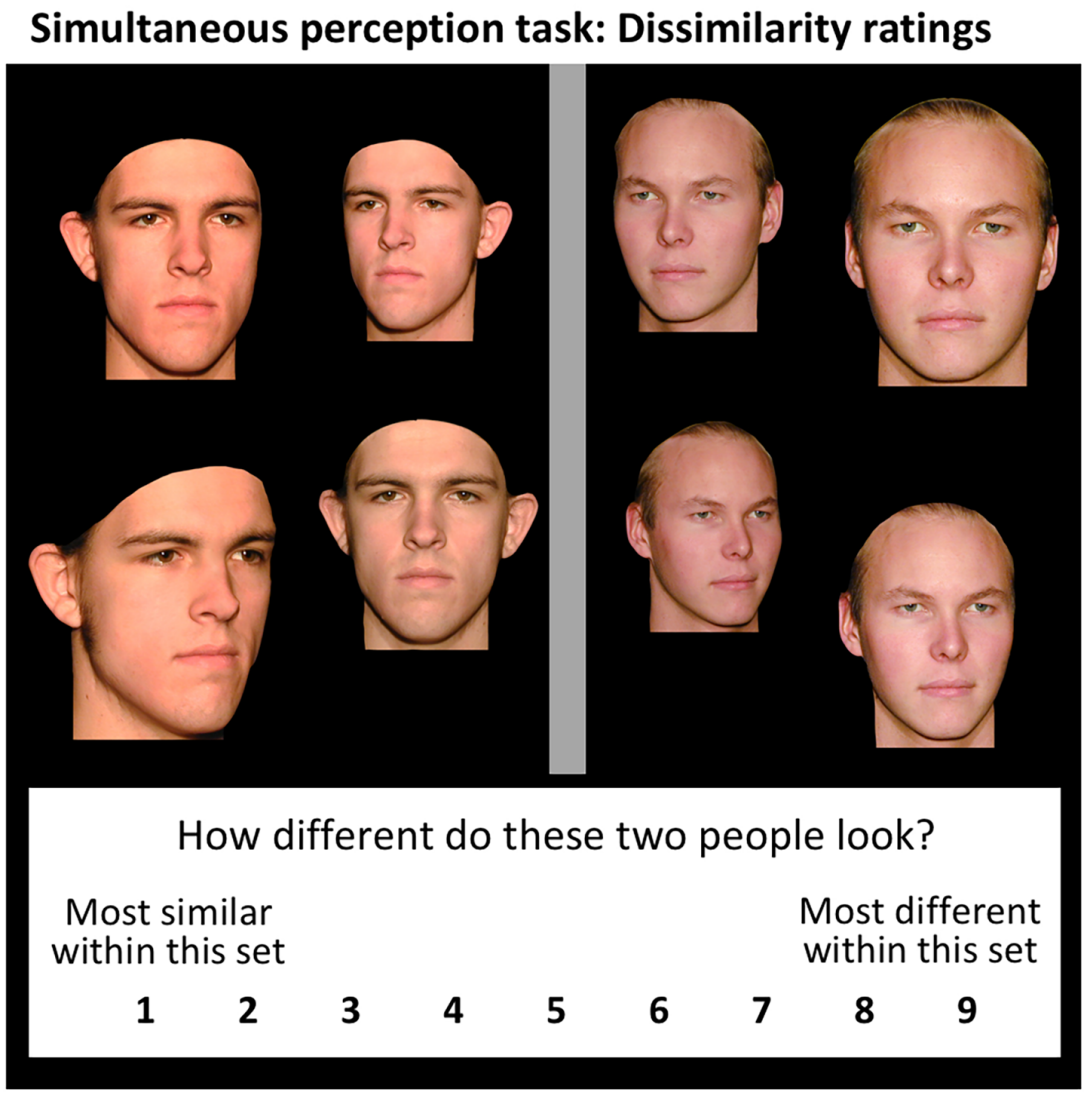
Example trial from our task. Two faces seen simultaneously captures the real-world situation in which a low-vision patient sees more than one person in a room at once who must be told apart. Dissimilarity ratings provide a sensitive measure of the degree to which the two identities appear different. Higher ratings indicate the two faces appear more different in identity (i.e., are easier to tell apart). Example trial is from high-resolution veridical condition in Experiment 1. Note the layout on the screen is not captured exactly, e.g., the face images were further apart horizontally.

To measure identity discrimination, we used the simultaneous perception task in Fig 4. This required rating 26 faces against each other for pairwise dissimilarity [8,9]. Given our previous findings that, using identical 147-point caricature stimuli to those we use here, low-resolution caricature improvements replicate well across multiple tasks including both perception and memory (dissimilarity ratings, old-new recognition, face-name learning) [8,9,23], our choice of task for the present study was based on efficiency of testing. In old-new recognition, our previous results [8,9] implied we would need of the order of 300 participants to complete experiments in the present article. In contrast, dissimilarity ratings produce smaller error bars, with a smaller number of observers, across a larger number of conditions, than either old-new recognition or face-name learning [8,9,23]. Using dissimilarity ratings allowed us to produce neat data on nine different conditions with only 40 participants in total.

On each trial, observers rated how dissimilar in identity two faces appeared, on a scale of 1 to 9. To the extent that caricaturing improves ability to tell apart faces, the relevant caricatured condition (C-68p or C-147p) will increase ratings of dissimilarity compared to veridical (V). Our specific research questions were: (a) Do we replicate our previous findings that high precision caricatures improve identity discrimination (i.e., predicting C-147p > V)? (b) Do less precise caricatures also produce a significant caricature benefit (i.e., predicting C-68p > V)? (c) How do less precise caricatures compare in effectiveness to highly precise caricatures (i.e., does C-68p remain below C-147p, or are these conditions equal)? and (d) Do the patterns revealed remain similar as resolution is decreased, or might it be, for example, that the less precise caricatures are effective at higher resolutions, but lose effectiveness as resolution worsens (e.g., mimicking prosthetic implants with fewer electrodes, or vision loss worsening over time in macular degeneration)?

## Method

### Participants

For Experiment 1, participants were N = 20 undergraduates (ages 18–22, mean = 19 yrs; 5 male, 15 female). For Experiment 2, participants were a different N = 20 undergraduates (ages 18–31, mean = 21 yrs; 7 male, 13 female). All were Caucasian, matching the race of the face stimuli, with normal or corrected-to-normal vision at the experimental screen-viewing distance of 60 cm (acuity of at least 20/20 using an ETDRS eye-chart). None reported conditions known to impair face perception (e.g., Autism Spectrum Disorder, prosopagnosia, brain injury). Participants were tested individually and given course credit or paid $15 for the 1–1.5 hour session (Experiment 1) or $20 for 1.5 hours (Experiment 2). The research followed the Declaration of Helsinki and was approved by Australian National University Human Research Ethics Committee (protocols 2015/305-2017a and 2015/305-2017b); informed written consent was obtained from subjects after explanation of the nature and possible consequences of the study.

### Stimuli

We used 26 faces (from [8]): 13 male and 13 female young adult Caucasians, with neutral expression, hair occluded, and no facial hair, glasses, etc. To ensure judgements were based on characteristics of the person’s *face*, not one specific photographic image, the task showed each person in four viewpoints (front-view, 10 degrees right, 10 degrees left, and 30 degrees left) and various sizes (4.3°–6.2° wide to 6.2°–9.1°, matching our previous blur and phosphenisation studies [8,9]). To ensure *identity*-specific information was caricatured, each image was caricatured away from an average face matched to the target for category (i.e., same race, sex, age-group, expression and viewpoint; e.g., a female face facing 30° left was caricatured away from an average 30° left female). Average faces were the average of a large number of individuals from that category (e.g., 50 young adult Caucasian males); for details see [8].

#### Creation of 147-point caricatures

The 147-point caricature stimuli were identical to those used in our previous studies [8,9,23]. The method for making them is detailed in full in [8]. Briefly, the 147 landmark points (see locations in Fig 3A) were placed by hand on every face, including the 104 Veridical images (26 identities x 4 viewpoints) and the 8 average faces (2 sexes x 4 viewpoints). Frontal views used the full 147 points; non-frontal views used 136 points due to only one ear showing. The caricatures were then created using Abrosoft FantaMorph 5 software. To avoid exaggerating lighting information in images (noting that lighting is not a reliable cue to identity), caricaturing was applied only to shape information not texture/reflectance (i.e., in morphing-software language, caricaturing was applied to the *warp* but not the *fade* function). The caricature strength was 60% (where 100% is defined as a doubling of distances between the landmark points in the veridical face and the average face); 60% was selected because our previous studies have found this produces maximum improvement in identity discrimination without introducing morphing artefacts (i.e., image discontinuities, such as a random slash across a part of the image) or making the face look weird [8,9] and thus falling outside the coding range of face-space [37].

#### Creation of 68-point caricatures

Yang et al [35] provide a full description of their automatic landmark point assignment procedure. Their software was applied to each of the 104 Veridical face images and 8 average faces, and the coordinates of the auto-assigned landmark points were saved. To ensure any differences in human perception of the 68-point and 147-point caricatures were due to the landmark number/locations (and thus to the precision of the caricatures), and not to other factors, we imported the 68-point coordinates into Abrosoft FantaMorph 5 and used identical caricaturing procedures for the 68-point caricatures as for the 147-point caricatures (i.e., both 60% strength, shape-only, caricatured away from sex-and-viewpoint-matched average, same average faces).

#### Experiment 1: Adding blur

For Experiment 1, we took the 312 high-resolution final images described above (104 Veridical, 104 C-68p, 104 C-147p), and rendered each in two levels of blur. This was designed to approximately simulate the blur seen by patients with macular degeneration, based on Marmor and Marmor’s [38] formula for blurring perceived in peripheral vision. Our labels Blur20 and Blur30 are as used in [8], where we had assumed peripheral viewing of a face subtending 18.11° along the horizontal (equivalent to a real person viewed at 54 cm [39]) at 20° eccentricity ('Blur 20' condition) or 30° eccentricity ('Blur 30' condition). These blur levels are not intended to be precise representations of the amount of blur seen by patients, but testing two levels of blur (Blur20 = moderate, Blur30 = severe) was used to reflect the general idea that, in patients, residual level of acuity varies widely in different individuals and at different stages of disease progression. Testing two blur levels also provides data on caricaturing benefits at two different levels of performance difficulty (noting that identity discrimination worsens as blur level increases [8]). The blurring formula applied uniform spatial blur across the image, by reducing the contrast of spatial frequencies higher than a given threshold (with threshold set lower for higher eccentricities, using a Gaussian kernel filter of size defined by the cutoff frequency;). Supporting Information S1 File provides more details of the blur procedure (also see [8]).

Note our simulation does not capture all aspects of macular degeneration in patients: as well as blur, some patients also report also seeing distortions or missing/moving parts [30]; patients vary in whether their residual acuity is supported by peripheral vision or by a small island of intact retina left within central vision; and while we used spatially uniform blur, the degree of blur across the face could vary depending on which region of their retina individual patients use to look at a face and the resolution of surrounding retinal regions. Note that simulating AMD precisely is an impossible task, given that every patient differs in their exact pattern of retinal damage, and also in their experience of how the world looks [30]. Our general argument is that, if similar caricature results are found across multiple blur levels, corresponding to looking at the face image at different distances into the periphery—and also in the very different phosphenised low-vision format of prosthetic vision simulation (Experiment 2)—then the results are not dependent on the precise details of the simulation, and thus are likely to transfer well to real patients.

#### Experiment 2: Adding phosphenisation

For Experiment 2, we took the 312 high-resolution final images described above (104 Veridical, 104 C-68p, and 104 C-147p), and rendered each in three different prosthetic vision resolutions. These were: a grid of 40x40 phosphenes tiling the face (hair-line to just below the chin) with all assumed to be functional (i.e., No Dropout, 40x40ND), a grid of 40x40 with a random 30% dropout of phosphenes (40x40DO) to simulate electrode failure or implantation of an electrode over dead retinal tissue (following [40]), and a grid of 32x32 with 30% random dropout (32x32DO). This range was chosen as based around resolutions similar to values realistic for some devices currently implanted in patients (e.g., 38 x 40 in [5]), while excluding lower resolutions at which we have previously found even 147-point caricaturing to be ineffective (i.e., 16x16DO in [8]). The phosphenisation followed the general simulation procedure used in many previous studies [8, 41–45]: each phosphene had a circular Gaussian intensity profile, and brightness at the nearest-neighbour image pixel to the electrode location was represented by a combination of size and centre-brightness of the phosphene, quantized to 8 discrete values. Supporting Information S1 File provides more details (also see [9]). Again, note our simulation procedure does not capture all aspects of visual appearance reported by prosthetic vision patients (e.g., distortion; details in [9]).

### Procedure

The task procedure followed Irons et al [8,9]. Each trial showed a pair of faces to be compared (Fig 2C), at a single resolution level and caricature value (e.g., if the face on the left was Blur20 and C68-p, then the face on the right was also Blur20 and C68-p). Male faces were compared only to male faces, and female to female, to test observers’ perception of *individual*-level identity. Given it would take too many trials to compare 13 males with all others, we split each sex into two subsets: one group of 7, and another of 6, and obtained dissimilarity ratings for each face rated against all other faces within the subset. For each experiment, this resulted in 648 trials per participant, comprising 72 trials (21 trials for 7 males compared to rest of 7-face subset + 15 trials for 6 males compared to rest of 6-face subset + repeat for females) in each of the 9 conditions (3 resolution levels x 3 caricature conditions).

Participants were encouraged to use the full 1–9 rating scale. In Experiment 1, trials were blocked by sex (with order counterbalanced across participants) but trials for all 9 conditions (3 blur x 3 caricature) were intermixed and shown in random order. In Experiment 2, trials were additionally blocked by resolution levels (in the order 40x40ND, then 40x40DO, then 32x32DO, for all participants) and, at the beginning of each new resolution block, participants were explicitly instructed to discard their previous criteria for use of the scale, and to start again to apply "most" and "least" similar within the set of faces for the new resolution. This was necessary because pilot testing revealed that mixing all three resolutions resulted in participants giving floor-level ratings for the lowest-resolution 32x32DO condition (i.e., rating all pairs as 1 or 2), meaning any differences between the caricature conditions (V, C-68, C-147) would be falsely flattened out and so could not be evaluated. Testing resolutions individually, and instructing participants to re-scale for each, lifted ratings off floor, and thus allowed fair comparison across the caricature conditions. A side effect of the rescaling instructions is that comparisons of overall ratings across resolutions are not valid in Experiment 2. (Note that where rescaling is not used, lower resolutions produce less perceived dissimilarity [9]).

Experiments were run in Superlab 4.5 on an iMac computer, with 27” flat screen (resolution 2560 x 1440).

### Scoring and preliminary analysis

For each participant, we calculated an average rating across all trials for each resolution and caricature condition. This was initially done separately for each sex of face, but preliminary 3 (resolution level) x 3 (caricature level) x 2 (sex of face) ANOVAs showed no main effects or interactions for sex (*p*s > .099 in Experiment 1, *p*s > .095 in Experiment 2), so scores were collapsed across male and female faces.

## Results

**Fig 5:**
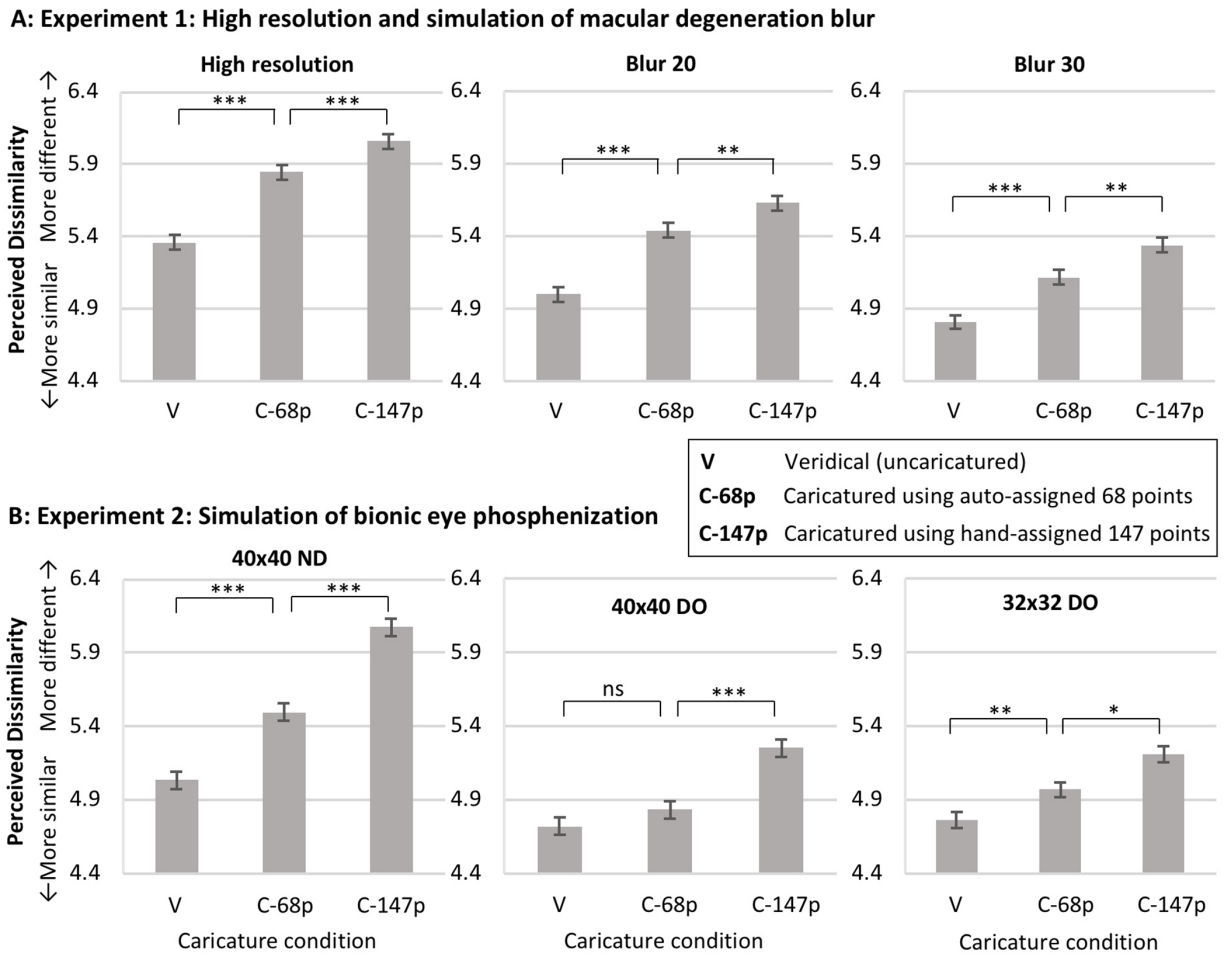
Results. Mean dissimilarity ratings in the simultaneous perception task, for **A**. Experiment 1 and **B**. Experiment 2, for the stimulus formats and resolutions illustrated in Fig 1. Higher ratings indicate that observers perceive greater difference between pairs of faces (i.e., greater differentiation in identity). The general pattern of findings is that: caricaturing improves identity differentiation relative to veridical; and caricatures based on auto-assigned 68 landmark points are effective (i.e., C-68p > V) although less effective than caricatures based on hand-assigned 147 landmark points (i.e., C68-p < C47-p). In Experiment 2, ratings cannot validly be compared across resolutions, because bionic eye resolutions were blocked and observers instructed to rescale their responses for each resolution (to avoid floor effects on caricature condition comparisons in lower-resolution conditions). Error bars are the equivalent of ±1SEM suitable for comparison across the caricature conditions, which are varied within-subjects (calculated as: MSE from a one-way ANOVA on caricature level, divided by square-root of N). *** = *p* < .001; ** = *p* < .01, * = *p* < .05, ns = *p* > .05. All t-tests paired samples, two-tailed.

Fig 5 shows the results. Statistical analysis targeted our specific research questions, and was thus *a priori*.

Concerning simple replication of our previous findings, in every resolution tested, for both blur (Experiment 1) and phosphenisation (Experiment 2), the 147-point caricatures significantly increased ratings of dissimilarity compared to the original veridical faces (i.e., *C-147p* > *V* for all 6 plots in Fig 5; all *t*s>5.47, all *p*s < .001). This replicates our previous findings [8,9] and confirms that our high precision caricatures, made from hand assignment of 147 landmark points, reliably increase the amount by which any two faces appear different in identity.

Our core issue then concerned the effectiveness of automatically assigned 68-point caricatures. Results showed, first, that the 68-point caricatures (*C-68p*) significantly improved identity differentiation, with higher dissimilarity ratings for *C-68p* than for veridical (*V*) for all resolution levels in our blur experiment (3 plots in Fig 5A; all *t*s>4.74, all *p*s < .001), and for the highest and lowest resolution levels in our bionic eye phosphenisation experiment (Fig 5B, t(19) = 6.14, *p* < .001 and t(19) = 3.62, *p* = .002 respectively; although not for the intermediate phosphenisation resolution, *t*(19) = 1.30, *p* = .208). Second, results showed that the 68-point caricatures did not reach the full effectiveness of the 147-point caricatures. Comparisons of *C-68p* with *C-147p* showed that, in all cases, dissimilarity ratings were significantly higher for *C-147p* than for *C-68p* (all 6 plots in Fig 5; all *t*s > 2.73, all *p*s < .013 and with 3 plots showing *p* < .001). Taken together, these two findings indicate that 68-point caricatures were significantly effective (i.e., improved identity perception relative to uncaricatured images), yet also significantly less effective than 147-point caricatures.

Concerning the question of whether the lower-precision caricatures might reduce in relative effectiveness at lower resolutions, there was no evidence of such a pattern. For blur (Experiment 1), Fig 5A shows no suggestion the C-68 condition gets any closer to V than to C-147 as resolution drops, and two-way ANOVA found no interaction between caricature condition and resolution, *F*(4, 76) = 2.02, *p* = .100. For phosphenisation (Experiment 2), two-way ANOVA did reveal a caricature condition x resolution interaction, *F*(4,76) = 13.18, *p* < .001, but Fig 5B shows this reflects primarily a reduced *overall* caricature effect at the lower resolutions (i.e., a reduced difference between V and 147-C; also see [9]), with the C-68 condition remaining approximately half-way between V and C-147 for both the highest resolution (40x40ND) and the lowest resolution (32x32DO).

We also computed a numerical estimate of the relative effectiveness of the 68-point caricatures compared to the 147-point caricatures, as *Relative Effectiveness* = [(*C-147p* − *V*) / (*C-68p* − *V*)] * 100. Overall, averaged across all 6 plots in Fig 5, this was 52%: that is, the 68-point caricatures were approximately half as effective at increasing identity differentiation as the 147-point caricatures. [Note: This calculation assumes observers’ use of the rating scale is linear with respect to their perception (e.g., that ratings of 4, 5, and 6 for *V*, *C-68p* and *C-147p* respectively would indicate that an observer perceived the increase in identity difference between face pairs to be half as strong for *C-68p* as for *C-147p*). While in general linearity of perception in rating scales cannot be guaranteed, in this case we think it is a reasonable assumption given ratings are well away from the compression expected at the ends of the scale range (i.e., with scale range 1–9, all ratings in Fig 5 fall between 4 and 6).]

Examining our different stimulus formats separately, relative effectiveness of 68-point caricatures was 68% for high resolution colour images, 65% for blurred faces (averaged across Blur20 and Blur30), and 39% for phosphenised faces (averaged across 40x40ND, 40x40DO and 32x32DO). Note that, while there is some suggestion here that the caricature benefit was stronger for blur (Experiment 1) than for phosphenisation (Experiment 2), the difference was not statistically significant *t*(37) = 1.97, *p* = .056.

## Discussion

Our results show that caricatures based on the auto-assigned 68-point landmarks of Yang et al [35] are significantly effective at improving identity perception, but less so than more precise 147-point caricatures. This was found across a wide range of face image types including high resolution faces, blurred faces simulating macular degeneration at two blur levels corresponding to moderate vision loss, and phosphenised faces simulating prosthetic vision at bionic eye resolutions ranging from values feasible in some current implants (32x32 grid with 30% random electrode dropout) up to values above the range of current technology (40x40 with all electrodes functional). Overall, the auto-assigned 68-point caricatures were approximately 50% as effective as caricatures based on hand-assignment of 147-point landmarks [8,9].

Concerning the ecological validity of the rating task used in the present study, we note that patients do not give explicit dissimilarity ratings to pairs of people they see in everyday life. However, they do commonly see more than one person simultaneously, and need to tell them apart. The more different they look—that is, the higher the dissimilarity rating the patient would give the two faces if asked—the easier they are to tell apart. We used ratings rather than, say, two-alternative-forced-choice same-different decision, because ratings provide a more fine-scaled measure of differences in identity perception. Thus, where we have found higher dissimilarity ratings, this directly implies that the caricatures being tested makes it easier to tell apart two (or more) faces seen in the relevant low-resolution format.

The rating task does not provide a direct measure of the amount by which caricaturing improves performance accuracy of face memory. However, there are strong reasons to believe that where our caricatures produced a significant increase in dissimilarity ratings, they will also produce a significant improvement in memory. First, it is well established that caricaturing improves recognition memory and identification (e.g., face naming) tasks. This includes in high resolution vision, with a variety of face caricature stimulus sets [8,17–23], and, most relevantly, also for the *identical low-vision simulations* and *identical 147-point caricature face stimuli* as we use here [8,9,23]. Moreover, increased dissimilarity and improved recognition memory are linked theoretically via the known properties of face-space: that is, shifting the face along a vector outwards from the centre moves it into a region of lower exemplar density with fewer confusable neighbours (thus improving old-new recognition, or face-naming accuracy, Fig 2), while also providing the best method of *maintaining its perceived identity as the same person* (e.g., outward shifts along the vector appear most like the veridical person, compared to morphs that produce sideways shifts within face-space, taking the face off the vector [46]).

A more open question is what fraction of the dissimilarity improvement for 68-point caricatures will translate to memory. If we assumed a linear transfer, then we would expect the relative effectiveness of 68-point caricatures to remain at approximately 50% of that of 147-point caricatures. Then, given that our 147-point caricatures produce an improvement in recognition accuracy of 6–14% [8,9,23] (e.g., if accuracy for veridical faces were 50% correct, accuracy for C-147p faces would improve to ~60% correct), the expected accuracy improvement for 68-point caricatures would be 3–7%. However, it is also possible the transfer to memory could be smaller or greater, for example if the rate at which the density of exemplars in face space reduces with increasing distance from the centre (which is a key factor driving improved memory for caricatures, Fig 2B) is not linear.

**Fig 6:**
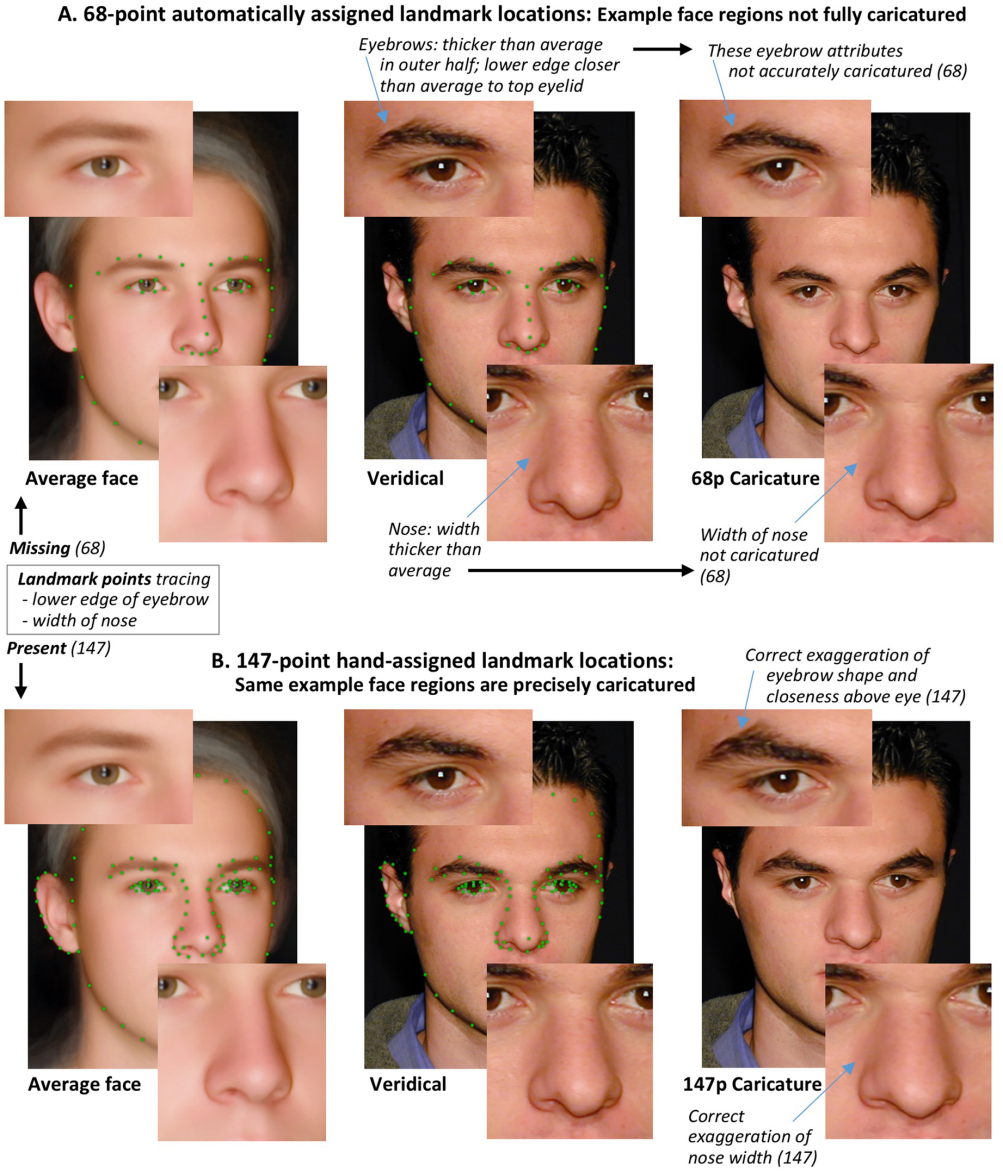
Why 68-landmark-point caricatures are less effective at improving identity differentiation. ** A**. The veridical male shown differs from the average face in several regions that are not coded by the 68-point landmarks, resulting in a lack of precise caricaturing of the eyebrows and nose (illustrated in expanded cutouts), and also the forehead and ears. **B**. These regions are precisely caricatured with the 147-point landmarks.

Concerning *why* 68-point caricatures are less effective, a key point is that this can be attributed only to the number/location of the landmark points and thus the precision of the resulting caricatures. This is because the 68-point caricatures and the 147-point caricatures were matched on all other variables (i.e., same caricature strength, same average-face images, same veridical face images, same caricaturing software and procedures; see Method). The 68-point landmarks leave various aspects of the face’s structure undescribed, or not described in full detail. When the caricaturing procedure is applied, some regions of the face then remain uncaricatured, or are not fully or exactly caricatured. For example, in Fig 6 (see further examples in S1 Fig) the veridical nose width is wider than average, but only the 147-point-caricature codes nose width and so correctly exaggerates this. In perceptual face-space, the fact that some regions of the face are being fully caricatured, while others are not, means the face will not be shifted directly away from the average on its original vector. Instead, the trajectory will be bent (with inflexion point at the veridical face) and, while the face will still be shifted further from the centre than when veridical, it will not necessarily best maintain its original identity [46].

Theoretically, our results imply that coding in humans’ perceptual face-space is very precise, using fine detail of exact shape of all regions of the face.

Practically, our results imply that the closer automatic landmark detection can get to replicating our hand-assigned 147-point locations, the better it will be for improving face recognition in low vision patients. Technology for landmark detection is progressing rapidly in deep learning approaches [47,48]; however, these generally require a large database of human-annotated face data to achieve state-of-the-art performance. The Yang et al [35] approach tested here operates without any human input, and extending that approach with more detailed landmark coding may be valuable, given its key abilities to locate faces and assign landmark points in real time to video input, and to cope with multiple face viewpoints and with partial face occlusion.

We also note there is potential for caricaturing to be applied more broadly than to face identity alone. With hand-assigned landmark points, face *expression* recognition can also be improved by caricaturing (exaggerating the expressive image of a particular person away from a neutral-face version of the same person), at least in high resolution stimuli [49]. (Note caricaturing is not suitable for improving object recognition in general, such as discriminating bicycles from chairs from dogs, because there is no "average object").

In conclusion, our overall research program has demonstrated that caricaturing—using high precision caricatures based on hand assignment of 147 landmark points—improves face identity perception under a wide range of circumstances relevant to patients, including telling apart two faces seen at once, recognising faces in memory tasks, two very different low-vision simulations, multiple resolution levels, a wide range of observer ages, and own- and other-race faces [8,9,23]. To translate caricaturing to everyday patient use, however, requires the ability to make caricatures automatically. The key take home message from our present results is that, although 68-point caricatures that can currently be created automatically do improve face identity perception, producing maximum improvement requires higher precision in assigning landmark points. Thus, an important direction for future research is to improve procedures in computer science, to increase the precision of auto-assignment of landmark points.

## Supporting information

S1 Fig Examples of 147-point vs 68-point caricatures. (PDF)

S1 File Supporting information. Details of blur technique used in Experiment 1. Details of Phosphenisation used in Experiment 2.
(PDF)

S2 File Data from the experiments. (XLSX)
