# Supplementary material for: Caricaturing faces to improve identity recognition in low vision simulations: How effective is current-generation automatic assignment of landmark points?
Source: PLoS One. 2018 Oct 4;13(10):e0204361. doi: 10.1371/journal.pone.0204361 (PMC6171855; doi:10.1371/journal.pone.0204361)
Supplement: S1 Fig — (PDF) [file pone.0204361.s001.pdf]

Caricaturing faces to improve identity recognition in low vision simulations: How effective is current-generation automatic assignment of landmark points?

Elinor McKone*, Rachel A. Robbins, Xuming He & Nick Barnes

* Corresponding author:

e-mail: Elinor.McKone@anu.edu.au (EM)

147-pt caricatures (60% exaggeration) | Veridical | 68-pt caricatures (60% exaggeration)

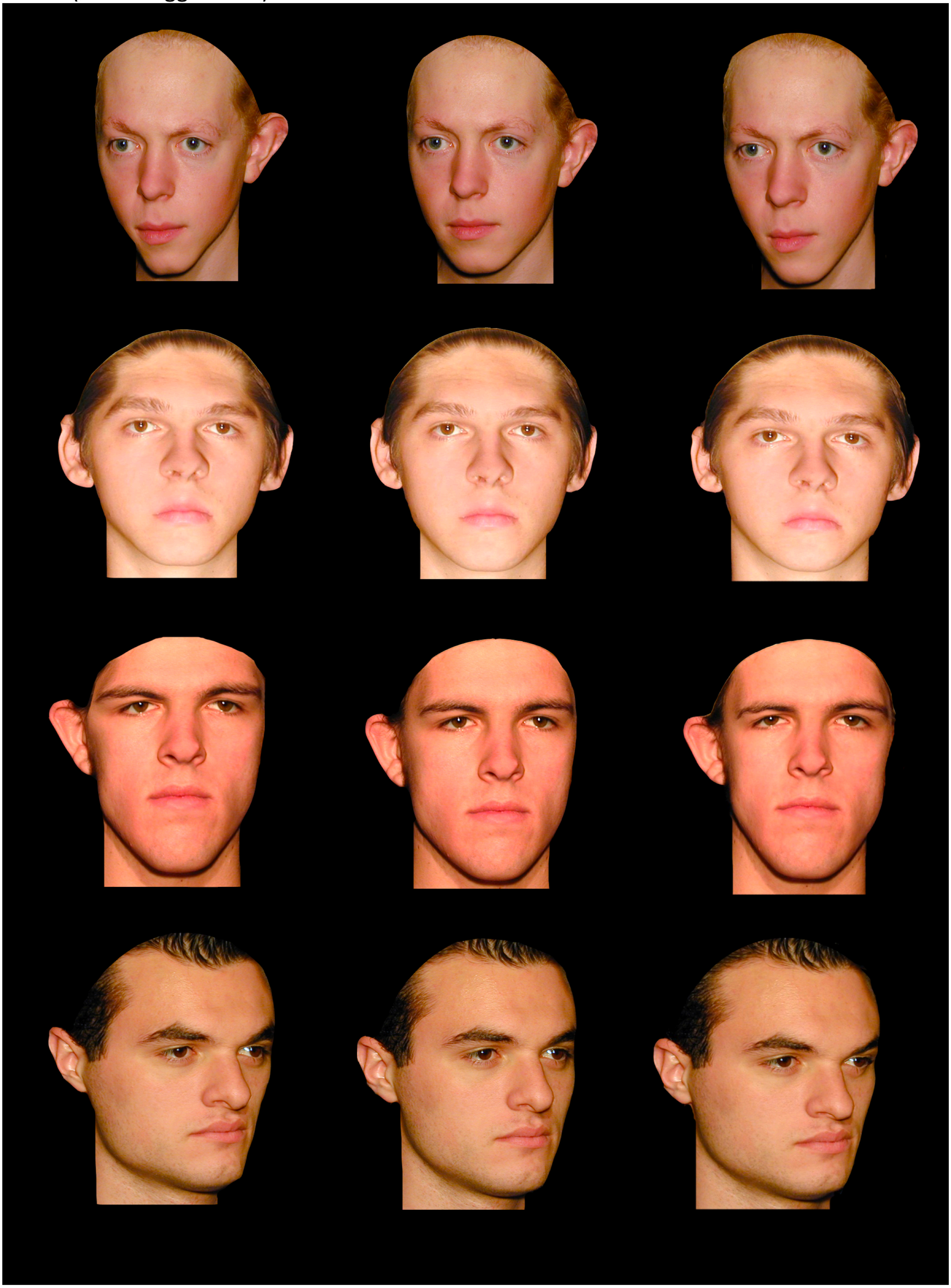

<u>S2 Fig</u>. Further examples of the 147-point vs 68-point caricatures. Examples selected to cover a range of identities and viewpoints. See main-text Figure 6 for explanation of the specific regions of the face where differences are most likely to emerge between the 147-point and 68-point versions.
