# Supplementary material for: Caricaturing faces to improve identity recognition in low vision simulations: How effective is current-generation automatic assignment of landmark points?
Source: PLoS One. 2018 Oct 4;13(10):e0204361. doi: 10.1371/journal.pone.0204361 (PMC6171855; doi:10.1371/journal.pone.0204361)
Supplement: S1 File — Details of blur technique used in Experiment 1. Details of Phosphenisation used in Experiment 2. (PDF) [file pone.0204361.s002.pdf]

S1 - Supporting Information

Caricaturing faces to improve identity recognition in low vision: How effective is current-generation automatic assignment of landmark points?

Elinor McKone, Rachel Robbins, Xuming He & Nick Barnes

January 2018

Details of blur technique used in Experiment 1

The peripheral blur simulation was implemented in the same way as in Irons et al. (2014). To quote details from that paper (pp. 9-10):

> "We simulate the acuity–eccentricity relation by removing the frequency components higher than the cutoff frequency at an eccentricity *e*. We implement this by applying a uniform spatial blur across the image using a Gaussian kernel filter of size defined by the cutoff frequency. It is well known that the cutoff frequency *f* (cpd) follows an inverse law with respect to the eccentricity (Anstis, 1974; Peli, Yang, & Goldstein, 1991; Rovamo, Virsu, & Näsänen, 1978). We represent this as $f = f_0/(1 + \beta e)$ and set the parameters as $\beta = 0.645$ and $f_0 = 30$ in this study. The resulting curve provides a close fit to the values presented in Marmor and Marmor (2010).
>
> Achieving theoretically ideal frequency cutoff using image filtering is difficult to implement without introducing artifacts; we adopt the conventional Gaussian filters with a kernel width σ_f as $(1/3)f$. This ensures that almost all the frequency components beyond *f* will be removed. Given the value of σ_f and the physical parameters used in the study, we convert the kernel width to pixel unit as follows. Let the width of screen be *w* cm, the distance between viewer and screen be *d* cm, and the horizontal resolution of the image be *r* pixels. Based on Fourier transform theory, the Gaussian kernel width in the spatial domain is $\sigma_s = 1/(2\pi\sigma_f)$ degrees. Therefore, we can compute the filter kernel width as $\sigma_s = 3(1 + \beta e)/2\pi f_0 \times r/2arctan(w/2d)$ pixel."

References

Anstis, S. M. (1974). Letter: A chart demonstrating variations in acuity with retinal position.

Vision Research, 14, 589–592. doi:10.1016/ 0042-6989(74)90049-2.
Peli, E., Yang, J., & Goldstein, R. E. (1991). Image variance with changes in size: The role of peripheral contrast thresholds. Journal of the Optical Society of America A, 8, 1762–1774. doi:10.1364/JOSAA.8. 001762.
Rovamo, J., Virsu, V., & Näsänen, R. (1978). Cortical magnification factor predicts the photopic contrast sensitivity of peripheral vision. Nature, 271, 54–56. doi:10.1038/271054a0.
Marmor, D. J., & Marmor, M. F. (2010). Simulating vision with and without macular disease. Archives of Ophthalmology, 128, 117–125. doi:10.1001/ archophthalmol.2009.366.
Irons, J., McKone, E., Dumbleton, R., Barnes, N., He, X., Provis, J., … Kwa, A. (2014). A new theoretical approach to improving face recognition in disorders of central vision: Face caricaturing. *Journal of Vision, 14*(2):12, 1–29, http:// www.journalofvision.org/content/14/2/12, doi: 10.1167/14.2.12.

Details of phosphenisation used in Experiment 2

REPRODUCED FROM SUPP MATIERALS OF IRONS ET AL (2017)

Supplementary Material: Face identity recognition in simulated prosthetic vision is poorer than previously reported and can be improved by caricaturing.

Jessica Irons, Tamara Gradden, Angel Zhang, Xuming He,
Nick Barnes, Adele Scott, Elinor McKone

January 2, 2017

1 Summary

We simulate prosthetic vision using a method adapted from Lieby et al. [2] and a framework similar to that described by Chen et al. [1]. The original image is processed according to the desired vision processing strategy. This processed image is sampled at each location in a given phosphene layout, which is typically a square or hexagonal lattice. Each sampled pixel intensity is quantised to each individual phosphene's dynamic range. We then simulate phosphenes as Gaussian profiles with size and brightness directly proportional to the quantised sampled pixel intensities. In addition, the brightness and size of each individual phosphene, relative to other phosphenes of the same intensity, can be specified. This is for example to simulate different sized electrodes. We refer to each phosphene profile as a "phosphene kernel" and each kernel is additively copied, up to saturation, to the final rendered image in the same layout as that used to sample the original image, with or without stochastic offsets added. We use the kernel caching method from Lieby et al. to improve rendering speed.

2 Details

Let

- $i \in [0, 1]$ be the sampled pixel intensity,
- $l > 0$ be the number of levels of intensity of the phosphene, e.g. 8 bits dynamic range $\implies l = 256$,
- $S > 0$ be the maximum phosphene size, which we fix to 0.357 multiplied by the spacing, in pixels, between phosphene centres in the rendered image[1],

[1]Setting the maximum phosphene size proportional to the phosphene spacing allows phosphene sizes to scale with changes in rendered image resolution or phosphene layout.

- $s > 0$ be the phosphene's relative size,
- $\max(s)$ be the maximum relative size across all phosphenes in the phosphene layout,
- $b > 0$ be the phosphene's relative brightness,
- $\max(b)$ be the maximum relative brightness across all phosphenes in the phosphene layout,
- $P[x, y] \in [0, 1]$ be the phosphene kernel.

Note that in this study, all relative phosphene sizes and brightnesses were set to 1, i.e. $s = b = 1$ for all phosphenes and $\max(s) = \max(b) = 1$.

The phosphene kernel is given by

$$P[x, y] = \frac{b}{\max(b)} I G(\sigma)[x, y] \tag{1}$$

where

$$I = \frac{[i(l-1)]}{l-1}, \tag{2}$$

$$G(\sigma)[x, y] = \begin{cases} e^{-\frac{x^2+y^2}{2\sigma^2}} & \text{if } |x|, |y| \leq \frac{w-1}{2}, \\ 0 & \text{otherwise,} \end{cases} \tag{3}$$

$$\sigma = \frac{s}{\max(s)} SI, \tag{4}$$

and

$$w = \begin{cases} \lfloor 6\sigma \rfloor & \text{if } \lfloor 6\sigma \rfloor \text{ odd,} \\ \lfloor 6\sigma \rfloor + 1 & \text{otherwise.} \end{cases} \tag{5}$$

The notation $[\cdot]$ in equation 2 denotes the rounding function.

3 Example implementation

Below is an example implementation of phosphene kernel calculation in MATLAB code.

```
%% Calculate a phosphene kernel
%
function P = calculate_phosphene_kernel(i, l, ...
                                         S, s, max_s, ...
                                         b, max_b)

    % Quantised sampled pixel intensity.
    I = round(i * (l - 1))/(l - 1);

    % Sigma
```

```
    sigma = s * S * I / max_s;

    % Kernel window size
    w = floor(6 * sigma);
    if(mod(w, 2) == 0)
        w = w + 1;
    end

    % Kernel
    [x, y] = meshgrid(-(w - 1)/2 : (w - 1)/2);
    P = b * I / max_b * exp(-(x.^2 + y.^2)/(2 * sigma^2));

end
```

4 References

[1] Chen, S., Suaning, G., Morley, J., and Lovell, N. (2009). Simulating prosthetic vision I. Visual models of phosphenes. *Vision Research, 49*(12):1493-1506.

[2] Lieby, P., Barnes, N., McCarthy, C., Liu, N., Dennett, H., Walker, J., Botea, V., and Scott, A. F. (2011). Substituting depth for intensity and real-time phosphene rendering: Visual navigation under low vision conditions. In *Engineering in Medicine and Biology Society, EMBC, 2011 Annual International Conference of the IEEE*, pp 8017-8020.
